# Exposure to point‐of‐sale displays and changes in susceptibility to smoking: findings from a cohort study of school students

**DOI:** 10.1111/add.12826

**Published:** 2015-01-20

**Authors:** Ilze Bogdanovica, Lisa Szatkowski, Ann McNeill, Dionysis Spanopoulos, John Britton

**Affiliations:** ^1^ UK Centre for Tobacco and Alcohol Studies, Division of Epidemiology and Public HealthUniversity of NottinghamClinical Sciences BuildingNottinghamUK; ^2^ UK Centre for Tobacco and Alcohol Studies, Addictions DepartmentInstitute of Psychiatry, King's College LondonLondonUK; ^3^ Division of Epidemiology and Public HealthUniversity of NottinghamClinical Sciences Building, City HospitalNottinghamUK

**Keywords:** Point‐of‐sale displays, smoking, smoking uptake, susceptibility, tobacco, youth

## Abstract

**Aims:**

To investigate the association between frequency of visiting shops and noticing of tobacco point‐of‐sale (PoS) displays and the development of susceptibility to smoking, or smoking uptake, in secondary school students.

**Design:**

Two surveys of a school based cohort study carried out in 2011 and 2012.

**Settings:**

Nottinghamshire, UK.

**Participants:**

A total of 2270 children aged 11–16 years from eight schools in Nottinghamshire.

**Measurements:**

We investigated changes in susceptibility to smoking and smoking status in relation to frequency of visiting shops and noticing PoS displays and number of tobacco brands recognized, controlling for a range of potential confounders. Susceptibility to smoking was defined using a set of three questions covering intentions to try smoking, to smoke within the next year and likelihood of smoking if a best friend offered a cigarette. For the analysis we used multinomial logistic regression.

**Findings:**

Among non‐susceptible never smokers, noticing PoS displays more frequently was associated independently with an increased risk of becoming susceptible to smoking [adjusted relative risk ratio (RRR) = 1.74; 99% confidence interval (CI) = 1.13–2.69], but was not associated with smoking uptake. Recognizing a higher number of brands among non‐susceptible never smokers doubled the risk of becoming susceptible to smoking and of becoming a smoker, but this did not have a significant effect on transition to smoking among susceptible never smokers. Frequency of noticing tobacco PoS displays was not associated significantly with smoking uptake among those who were susceptible never smokers at baseline.

**Conclusions:**

Noticing tobacco point‐of‐sale displays more often and recognizing a higher number of tobacco brands is associated with an increased risk of becoming susceptible to smoking among adolescents in the United Kingdom, and recognizing a higher number of brands is associated positively with an increased risk of smoking uptake.

## Introduction

Smoking is the largest preventable cause of death in industrialized countries, and in the United Kingdom accounts for more than 100 000 deaths every year 1. Because the majority of deaths from smoking occur in people who became regular smokers during adolescence, preventing young people from initiating smoking and becoming regular smokers is a clear public health priority.

Of the many causes of smoking initiation in adolescence, exposure to tobacco advertising and promotion are important, as they are entirely preventable 2, 3, 4. In the United Kingdom, most forms of tobacco advertising and promotion are now prohibited under the terms of the 2002 Tobacco Advertising and Promotion Act 5 and, as a result, the tobacco industry has increased the use of forms of advertising and promotion not covered by the Act. These include point‐of‐sale (PoS) displays and the tobacco pack itself, both of which promote tobacco brands to existing and new customers 6, 7. Exposure of existing smokers to tobacco products in PoS displays increases the likelihood of purchasing 8, makes quitting more difficult by urging recent quitters to smoke 9, and although there is less evidence of effects on potential new smokers, cross‐sectional data suggest that adolescents who recall PoS exposure are more likely to be smokers or to be susceptible to smoking uptake 10, 11. Most smokers take up smoking during adolescence. Susceptibility to smoking, defined as absence of a firm decision not to smoke 12, has been shown to be a strong marker of experimentation with and uptake of smoking among adolescents 13, 14. There is also evidence that exposure to tobacco advertising increases susceptibility to smoking 12, and therefore potentially smoking uptake. Tobacco PoS displays placed in shops visited by children is an effective way to communicate brand imagery 15. Consistent with this observation, the prevalence of smoking tends to be higher in schools with a higher density of tobacco outlets and in‐store promotion of tobacco products in their surrounding area 16, 17. Recent findings from a study carried out in Australia suggest that removal of PoS displays has contributed to de‐normalization of smoking among young people, and has led to a decrease in brand awareness and overestimation of peer smoking 18. Some evidence from experimental studies confirms that removal of open PoS displays could prevent young people from attempting to purchase tobacco products 19. However, while countries including Ireland, Norway, Australia, Finland and New Zealand have now prohibited PoS tobacco displays, evidence of the impact of prohibition on smoking behaviour remains limited.

In England, open PoS displays are being prohibited in two stages, starting with large shops such as supermarkets from April 2012, and in smaller retailers, which occur typically in the locality of schools and are the main source of children's exposure to PoS displays 11, from April 2015. We have investigated prospectively the association between PoS exposure and the development of susceptibility to smoking, and uptake of smoking, among secondary school students in the period leading up to the first stage of PoS prohibition in April 2012.

## Methods

### Data collection

In March 2011, we carried out a cross‐sectional study of smoking and PoS display exposure in students attending 11 secondary schools in Nottingham 11. We then invited the same 11 schools to repeat the survey in March 2012, immediately before the English law prohibiting PoS displays in large retailers came into force, and eight schools agreed to do so. Informed consent was obtained from the head teachers of all participating schools, and opt‐out consent forms distributed to parents of children in school years 7–11 [aged 11–16]. Ethics approval for data collection was granted by the University of Nottingham School of Education Research Ethics Committee.

All students whose parents did not decline consent were invited to complete a paper‐based questionnaire, under teacher supervision, during the school day. The questionnaire collected data on age, sex, postcode [from which quintiles of the Index of Multiple Deprivation (IMD) were derived as an area‐level measure of socio‐economic status 20], rebelliousness (by asking whether a student gets into trouble in school, does things their parents would not want to them to do and likes scary and dangerous things, and split into two categories—high versus low levels of rebelliousness—based on the median value) and self‐perceived academic performance (self‐reported evaluation of grades). We also included questions on smoking among family members and friends, and whether smoking was allowed in the main family home. Smoking status was ascertained using questions based on the national ‘Smoking, Drinking and Drug Use among Young People in England’ survey questionnaire 21. Never smokers were defined as those who reported that they had never smoked, not even a puff or two; all who had tried smoking or were current smokers were defined as ever smokers. Smoking susceptibility among never smokers was categorized using three previously validated questions 22: ‘Do you think you will try a cigarette soon?’ (yes/no); ‘If one of your best friends were to offer you a cigarette would you smoke it’ (definitely yes/probably yes/probably not/definitely not); and ‘Do you think you will smoke a cigarette at any time during next year?’ (definitely yes/probably yes/probably not/definitely not). Those who answered ‘no’ to the first question and ‘definitely not’ to the following two questions were classified as non‐susceptible, and any other combination as susceptible to smoking 22.

We measured the frequency of visiting small shops and supermarkets by asking students how often they go to each of these categories of shops (almost every day, two or three times a week, once a week, two or three times a month, once a month, less than once a month), and merging these responses into a binary variable—fewer than two or three times a week, or two or three times a week or more—to avoid categories with small numbers. We measured frequency of noticing tobacco PoS displays by asking students whether, when going to shops, they noticed cigarettes on display every time, most times, sometimes, hardly ever or never. We merged these categories into a binary variable comprising sometimes or less, and most or every time to avoid categories with small numbers. We examined the brands of cigarettes and hand‐rolling tobacco that students recognized by listing the most popular brands (based on frequency of display in shops in Nottingham 23) in these categories and asking respondents to indicate all brands they noticed when visiting supermarkets or small shops. We grouped the total number of brands recognized in our analysis into three categories (none, one to five brands and more than five brands, split at the median value after excluding those who reported ‘none’).

### Analysis

We used students' forenames, surnames, school and school year to link data for individual students in years 7–10 in 2011 and years 8–11 in 2012 to investigate changes in susceptibility and smoking status, manually checking cases we were not able to match for spelling changes or data entry errors. Four outcome variables were defined: (1) susceptibility to smoking in 2012 among students who were non‐susceptible never smokers in 2011; (2) ever smoking in 2012 among students who were non‐susceptible never smokers in 2011; (3) non‐susceptibility to smoking in 2012 among students who were susceptible never smokers in 2011 and (4) ever smoking in 2012 among students who were susceptible never smokers in 2011. Our main exposure variables were frequency of visiting shops in 2011, noticing of tobacco PoS displays in 2011 and number of brands recognized in 2011, with adjustment for suspected confounders using data from 2011. Additionally, we used two combined exposure variables to estimate joint effects: frequency of visiting shops combined with frequency of noticing PoS displays, and frequency of noticing tobacco PoS displays combined with number of brands recognized. Students with missing values for outcome variables were excluded from the analysis, but those with missing exposure data were included, coding missing values as a separate category, to maximize study power.

We used multinomial logistic regression to obtain relative risk ratios (RRRs) for changes in smoking and susceptibility status relative to no change between 2011 and 2012 in children who are exposed frequently to PoS displays, noticed PoS displays more often and recalled higher number of brands, compared to children who did not report these exposures. We also investigated the association between the combined exposure variables and changes in smoking status. We first built two unadjusted multinomial models, one restricted to those who were non‐susceptible never smokers at baseline and a second restricted to those who were susceptible never smokers at baseline, and then adjusted these models for potential confounding variables that were found to be significant at univariable level. Likelihood ratio tests were used to determine which of these confounding variables should be included in the final models. Given the large number of statistical tests carried out, we present 99% confidence intervals (CIs) for each measure of association, as well as exact *P*‐values with significance levels set at 0.01. The students who responded to our survey are clustered within classes and schools, and thus we needed to account for this non‐independence in our analysis. However, the small total number of students (particularly baseline susceptible never smokers), and students per cluster, meant that we could not fit a multi‐level model. Therefore, for all models we used a clustered sandwich estimator to produce robust 99% confidence intervals around our point estimates of effect to account for the clustering.

It is possible that the inclusion of parental, sibling and friend smoking as confounding variables may lead to over‐adjustment, as these variables may themselves be related to exposure to tobacco marketing. Therefore, as a sensitivity analysis we built adjusted models where these variables were not considered as potential confounders.

Data were analysed using Stata 13 (Stata Corporation, College Station, TX, USA).

## Results

We received questionnaires from 4302 students (approximately 69% of those eligible, based on the total number of students reported by schools to be on their rolls) from the eight schools surveyed in 2012, of whom 3672 were in school years 8–11 and hence potentially also participants in the 2011 survey 11. We were able to link questionnaires from 2011 and 2012 for 2354 (64%) of these, but had to exclude 47 respondents who did not provide data on susceptibility to smoking in both years, and 37 respondents with incompatible primary outcome responses (24 who reported in 2012 that they were non‐susceptible never smokers, having been ever smokers in 2011; and 13 who indicated that they were susceptible never smokers in 2012, having been ever smokers in 2011). We were therefore able to track smoking and susceptibility status over time in 2270 respondents who, at baseline, comprised 1576 non‐susceptible never smokers, 494 susceptible never smokers and 200 ever smokers.

Of the non‐susceptible never smokers in 2011, 313 (19.9%) became susceptible never smokers in 2012 and 111 (7.0%) became ever smokers. Of the 494 susceptible never smokers in 2011, 224 (45.3%) did not change status, while 128 (25.9%) became non‐susceptible never smokers and 142 (28.7%) progressed to being a smoker. Other characteristics of the students included in the analysis are presented in Table 1.

**Table 1:** Summary of 2011 and 2012 data for the 2270 participants with linked responses.

*Variable*	*2011 (number, %)*	*2012 (number, %)*
Sex		
Boy	1120 (49.3)	1120 (49.3)
Girl	1150 (50.7)	1150 (50.7)
Age (years)		
11	261 (11.5)	
12	672 (29.6)	257 (11.3)
13	668 (29.4)	698 (30.8)
14	511 (22.5)	660 (29.1)
15	149 (6.6)	501 (22.1)
16		147 (6.5)
Missing	9 (0.4)	7 (0.3)
Deprivation quintile		
1 (least deprived)	757 (33.4)	641 (28.2)
2	288 (12.7)	259 (11.4)
3	354 (15.6)	330 (14.5)
4	300 (13.2)	289 (12.7)
5 (most deprived)	283 (12.5)	271 (11.9)
Missing	288 (12.7)	480 (21.2)
Parental smoking		
Neither parent smokes	1580 (69.6)	1581 (69.7)
One parent smokes	460 (20.3)	456 (20.1)
Both parents smoke	209 (9.2)	190 (8.4)
Missing	21 (0.9)	43 (1.9)
Sibling smoking		
None smokes	2062 (90.8)	1980 (87.2)
At least one smokes	187 (8.2)	247 (10.9)
Missing	21 (0.9)	43 (1.9)
Smoking in the main family home		
Not allowed	1845 (81.3)	1914 (84.3)
Allowed	395 (17.4)	312 (13.7)
Missing	30 (1.3)	44 (1.9)
Number of smoking friends		
None	1117 (49.2)	702 (30.9)
One or two	276 (12.2)	340 (15.0)
Three or more	350 (15.4)	628 (27.7)
Not sure	498 (21.9)	557 (24.5)
Missing	29 (1.3)	43 (1.9)
Self‐perceived academic performance		
Excellent or good	1787 (78.7)	1686 (74.3)
Average or below average	448 (19.7)	544 (24.0)
Missing	35 (1.5)	40 (1.8)
Rebelliousness		
Low	1253 (55.2)	1263 (55.6)
High	956 (42.1)	906 (39.9)
Missing	61 (2.7)	101 (4.5)
Susceptibility to smoking		
Non‐susceptible never smoker	1576 (69.4)	1280 (56.4)
Susceptible never smoker	494 (21.8)	537 (23.7)
Ever smoker	200 (8.8)	453 (20.0)
Notice cigarettes on displays		
Sometimes or less	442 (19.5)	436 (19.2)
Most times or every time	1825 (80.4)	1796 (79.1)
Missing	3 (0.1)	38 (1.7)
Frequency of visiting shops		
Fewer than 2 or 3 times a week	824 (36.3)	871 (38.4)
At least 2 or 3 times a week	1444 (63.6)	1386 (61.1)
Missing	2 (0.1)	13 (0.6)
Number of brands recognized		
None	650 (28.6)	547 (24.1)
1–5 brands	809 (35.6)	754 (33.2)
More than 5 brands	556 (24.5)	759 (33.4)
Missing	255 (11.2)	210 (9.3)

### Change in smoking susceptibility and status in relation to exposure variables at univariable level

Among those who were non‐susceptible never smokers in 2011, the univariable RRRs of becoming susceptible to smoking in 2012 compared to remaining non‐susceptible were significantly higher among students with parents who smoked, or with more friends who smoked, among those with lower perceived levels of academic performance and higher levels of rebelliousness, those who visited shops more frequently and noticed cigarettes on PoS displays more often, and those who recognized a higher number of brands (Table 2).

**Table 2:** Unadjusted relative risk ratios for changes in susceptibility and smoking status in relation to explanatory variables.

	*Among non‐susceptible never smokers at baseline*						*Among susceptible never smokers at baseline*					
	*RRR of becoming susceptible*			*RRR of becoming an ever smoker*			*RRR of becoming non‐susceptible*			*RRR of becoming an ever smoker*		
	*Estimate*	*99% CI*	*P*	*Estimate*	*99% CI*	*P*	*Estimate*	*99% CI*	*P*	*Estimate*	*99% CI*	*P*
Sex												
Boy	1.00			1.00			1.00			1.00		
Girl	1.12	0.87–1.43	0.242	1.47	0.89–2.45	0.049	0.53	0.23–1.25	0.056	1.45	1.16–1.81	<0.001
Age (years)												
11	1.00			1.00			1.00			1.00		
12	1.10	0.51–1.96	0.985	1.81	0.42–7.84	0.296	0.49	0.20–1.22	0.043	0.38	0.16–0.90	0.004
13	1.32	0.66–2.65	0.304	4.12	1.71–9.97	<0.001	0.26	0.11–0.60	0.000	0.54	0.23–1.24	0.057
14	1.11	0.55–2.26	0.697	4.64	1.49–14.5	0.001	0.55	0.28–1.08	0.023	1.26	0.40–3.96	0.610
15	0.52	0.15–1.80	0.174	8.51	2.18–33.1	<0.001	0.39	0.15–1.01	0.011	0.67	0.32–1.42	0.172
Quintile of index of multiple deprivation												
1 (least deprived)	1.00			1.00			1.00			1.00		
2	1.10	0.60–2.02	0.686	1.31	0.46–3.77	0.507	0.42	0.21–0.81	0.001	0.98	0.51–1.90	0.946
3	1.08	0.59–1.99	0.736	1.33	0.68–2.59	0.279	1.01	0.48–2.12	0.965	0.94	0.42–2.13	0.854
4	0.79	0.43–1.45	0.326	0.70	0.25–1.95	0.373	0.91	0.24–3.43	0.852	1.43	0.27–7.68	0.581
5 (most deprived)	1.16	0.60–2.22	0.564	1.28	0.53–3.14	0.471	0.58	0.17–2.00	0.258	1.15	0.58–2.25	0.602
Parental smoking												
Neither parent smokes	1.00			1.00			1.00			1.00		
One parent smokes	1.57	1.08–2.20	0.002	2.62	0.94–7.26	0.015	1.48	0.86–2.55	0.060	1.63	0.98–2.70	0.013
Both parents smoke	1.10	0.49–2.45	0.771	3.33	1.37–8.11	<0.001	1.08	0.37–3.15	0.846	2.48	0.90–6.85	0.022
Sibling smoking												
None smokes	1.00			1.00			1.00			1.00		
At least one smokes	1.76	0.74–4.14	0.091	1.54	0.58–4.08	0.256	1.10	0.60–2.03	0.682	1.83	0.94–3.57	0.019
Smoking in the main family home												
Not allowed	1.00			1.00			1.00			1.00		
Allowed	1.49	0.91–2.45	0.037	2.50	1.34–4.65	<0.001	1.06	0.42–2.68	0.860	1.47	0.70–3.08	0.181
Number of friends who smoke												
None	1.00			1.00			1.00			1.00		
One or two	1.61	0.85–3.07	0.055	1.94	0.61–6.13	0.139	0.62	0.22–1.76	0.234	1.45	0.92–2.30	0.037
Three or more	1.66	1.40–1.96	<0.001	4.50	1.40–14.5	0.001	0.56	0.33–0.96	0.005	2.33	1.33–4.07	<0.001
Not sure	1.94	1.34–2.83	<0.001	3.65	1.50–8.90	<0.001	0.89	0.39–2.02	0.705	1.79	1.22–2.62	<0.001
Self‐perceived academic performance												
Excellent or good	1.00			1.00			1.00			1.00		
Average or below average	1.84	1.16–2.92	0.001	2.25	1.20–4.21	0.001	0.72	0.26–1.94	0.389	0.92	0.39–2.15	<0.001
Rebelliousness												
Low	1.00			1.00			1.00			1.00		
High	1.60	1.09–2.33	0.001	2.58	1.52–4.35	<0.001	1.17	0.71–1.95	0.419	1.33	0.78–2.26	0.164
Noticing point‐of‐sale displays												
Sometimes or less	1.00			1.00			1.00			1.00		
Most or every time	1.80	1.12–2.88	0.001	2.15	0.96–4.82	0.014	2.10	0.78–5.64	0.053	1.17	0.47–2.93	0.664
Frequency of visiting shops												
Fewer than 2 or 3 times a week	1.00			1.00			1.00			1.00		
At least 2 or 3 times a week	1.52	1.16–1.99	<0.001	1.75	0.91–3.34	0.026	1.20	0.64–2.23	0.461	1.49	0.88–2.52	0.053
Number of brands recognized												
None	1.00			1.00			1.00			1.00		
1–5	1.92	1.25–2.94	<0.001	1.60	1.06–2.40	0.003	0.88	0.36–2.14	0.715	1.22	0.70–2.15	0.357
More than 5	2.31	1.62–3.29	<0.001	2.93	1.88–4.57	<0.001	0.91	0.48–1.73	0.700	1.96	1.19–3.23	0.001
Missing	2.81	1.78–4.43	<0.001	1.35	0.48–3.81	0.453	1.27	0.34–4.67	0.641	1.56	0.47–5.20	0.342
Combined frequency of visiting and noticing displays												
Visit <2/3 times per week/notice sometimes or less	1.00			1.00			1.00			1.00		
Visit <2/3 times per week/notice most or every time	2.72	1.32–5.58	<0.001	1.95	0.33–11.5	0.331	1.83	0.47–7.07	0.250	0.66	0.21–2.09	0.350
Visit >2/3 times per week/notice sometimes or less	2.71	1.20–6.09	0.002	1.58	0.21–11.6	0.555	0.90	0.13–6.42	0.888	0.75	0.21–2.61	0.550
Visit >2/3 times per week/notice most or every time	3.53	1.69–7.38	<0.001	3.25	0.74–14.2	0.040	2.07	0.41–1.04	0.244	1.18	0.37–3.84	0.710
Combined frequency of noticing displays and brand recognition												
Notice sometimes or less/0 brands	1.00			1.00			1.00			1.00		
Notice sometimes or less/1–5 brands	2.05	0.82–5.16	0.045	1.53	0.20–11.8	0.590	0.88	0.13–5.73	0.855	1.05	0.38–2.88	0.901
Notice sometimes or less/6+ brands	2.53	0.67–9.51	0.072	1.53	0.11–22.3	0.681	*			1.26	0.16–9.80	0.772
Notice most or every time/0 brands	1.76	0.63–4.88	0.155	1.66	0.45–6.12	0.321	2.84	0.70–11.6	0.055	0.92	0.25–3.43	0.868
Notice most or every time/1–5 brands	2.93	1.06–8.09	0.006	2.37	0.81–6.92	0.037	2.01	0.51–7.88	0.187	1.18	0.29–4.85	0.761
Notice most or every time/6+ brands	3.47	1.29–9.33	0.001	4.34	1.66–11.4	<0.001	2.09	0.58–7.46	0.137	1.92	0.59–6.19	0.153
Missing	4.20	1.40–12.6	0.001	1.90	0.70–5.17	0.098	2.68	0.63–11.4	0.080	1.48	0.32–6.90	0.510

* Could not estimate due to small numbers. RRR = relative risk ratio; CI = confidence interval.

Also among non‐susceptible never smokers in 2011, the univariable RRRs of having become an ever smoker in 2012 compared to remaining non‐susceptible were higher with increasing age among those whose parents smoke, from families where smoking was allowed in the main home, those with a greater number of smoking friends, with lower levels of academic achievement or higher levels of rebelliousness, among those who recognized more brands (Table 2).

Among susceptible never smokers in 2011 the univariable RRRs of reporting non‐susceptibility in 2012 compared to persisting susceptibility were lower in older age groups and in those with more friends who smoked, but did not show significant associations with any other variable. Among susceptible never smokers in 2011 the univariable RRRs of becoming an ever smoker in 2012 compared to remaining susceptible were higher among girls, with a greater number of smoking friends, with lower levels of academic achievement, and those who recognized higher number of brands, but not in relation to visiting shops or noticing PoS displays (Table 2).

### Change in smoking susceptibility and status in relation to exposure variables at multivariable level

After adjustment for confounding variables, non‐susceptible never smokers at baseline who visited shops and noticed PoS displays more frequently, and who recognized more brands, were more likely to become susceptible than respondents without these exposures (Table 3). Non‐susceptible never smokers who recognized more than five brands were approximately twice as likely to become ever smokers compared to those who recognized no brands (adjusted RRR = 2.12, 99% CI = 1.64–2.75, *P* < 0.001). There was no association between frequency of visiting shops and noticing PoS displays and progression to smoking among baseline non‐susceptible never smokers.

**Table 3:** Adjusted relative risk ratios for changes in susceptibility and smoking status in relation to noticing point‐of sale (PoS) displays, frequency of visiting shops and number of brands recognized.

	*Among non‐susceptible never smokers at baseline*						*Among susceptible never smokers at baseline*					
	*RRR of becoming susceptible* a			*RRR of becoming an ever smoker* a			*RRR of becoming non‐susceptible* b			*RRR of becoming an ever smoker* b		
	*Estimate*	*99% CI*	*P*	*Estimate*	*99% CI*	*P*	*Estimate*	*99% CI*	*P*	*Estimate*	*99% CI*	*P*
Noticing point of sale displays												
Sometimes or less	1.00			1.00			1.00			1.00		
Most or every time	1.74	1.13–2.69	0.001	1.93	0.89–4.18	0.028	2.12	0.88–5.11	0.028	1.31	0.53–3.21	0.438
Frequency of visiting shops												
Fewer than 2 or 3 times a week	1.00			1.00			1.00			1.00		
At least 2 or 3 times a week	1.32	1.04–1.67	0.002	1.32	0.62–2.79	0.341	1.17	0.65–2.11	0.492	1.49	0.91–2.45	0.039
Number of brands recognized												
None	1.00			1.00			1.00			1.00		
1–5	1.83	1.24–2.70	<0.001	1.34	0.71–2.55	0.237	0.91	0.31–2.70	0.823	1.06	0.57–1.98	0.808
More than 5	2.16	1.68–2.78	<0.001	2.12	1.64–2.75	<0.001	0.76	0.37–1.53	0.311	1.65	0.88–3.09	0.038
Combined frequency of visiting and noticing displays												
Visit <2/3 times per week/notice sometimes or less	1.00			1.00			1.00			1.00		
Visit <2/3 times per week/notice most or every time	2.63	1.30–5.30	<0.001	1.90	0.35–10.3	0.329	1.76	0.47–6.61	0.272	0.75	0.25–2.24	0.500
Visit >2/3 times per week/notice sometimes or less	2.92	1.04–5.05	0.007	1.29	0.17–9.89	0.746	0.82	0.10–6.55	0.806	0.75	0.27–2.07	0.458
Visit >2/3 times per week/notice most or every time	3.00	1.38–6.53	<0.001	2.38	0.47–12.1	0.170	2.00	0.46–8.65	0.225	1.33	0.45–3.93	0.502
Combined frequency of noticing displays and brand recognition												
Notice sometimes or less/0 brands	1.00			1.00			1.00			1.00		
Notice sometimes or less/1–5 brands	1.97	0.82–4.70	0.046	1.43	0.18–11.1	0.652	0.89	0.10–7.68	0.892	1.00	0.28–3.59	0.995
Notice sometimes or less/6+ brands	2.32	0.70–7.63	0.069	1.14	0.06–23.3	0.911	*			0.84	0.12–5.90	0.822
Notice most or every time/0 brands	1.74	0.69–4.43	0.124	1.88	0.50–7.09	0.222	3.12	0.78–12.4	0.034	1.11	0.40–3.12	0.794
Notice most or every time/1–5 brands	2.73	1.13–6.61	0.003	2.16	0.61–7.61	0.116	2.20	0.60–8.10	0.120	1.15	0.31–4.35	0.781
Notice most or every time/6+ brands	3.23	1.45–7.17	<0.001	3.42	1.26–9.31	0.002	1.82	0.55–5.99	0.197	1.86	0.59–5.81	0.163

a Adjusted for age, sex, parental smoking, friend smoking, self‐perceived academic performance and rebelliousness;

b adjusted for age, sex and parental smoking;

* could not estimate due to small numbers. RRR = relative risk ratio; CI = confidence interval.

Based on 99% CIs there were no significant associations between frequency of visiting shops, noticing displays and brand recognition and changes in smoking status among students who were susceptible never smokers at baseline.

When frequency of visiting shops was combined with frequency of noticing PoS displays, increases in the risk of non‐susceptible never smokers becoming susceptible were seen across all categories compared to those who both visit shops and notice PoS displays infrequently. Non‐susceptible never smokers who noticed PoS displays most or every time, and who recognized at least one brand, were approximately three times more likely to become susceptible compared to those who infrequently noticed PoS displays and recognized no brands. Non‐susceptible never smokers who noticed PoS displays most or every time and who recognized more than five brands were more likely to have progressed to smoking by 2012 (adjusted RRR = 3.42, 99% CI = 1.26–9.31, *P* = 0.002).

The results of sensitivity analyses excluding from the list of potential confounders parental, sibling and friend smoking which may, themselves, be related to tobacco marketing, are presented as Supporting information (Table S1). Here, the previously significant associations between noticing point of sale displays and changes in smoking status among baseline non‐susceptible never smokers (to both susceptible never smokers and ever smokers) are now non‐significant. However, susceptible never smokers in 2011 who recognized more than five brands were now significantly more likely to have progressed to smoking in 2012, with a 99% CI that excludes the possibility of no association (adjusted RRR = 2.08, 99% CI = 1.30–3.34, *P* < 0.001).

## Discussion

We have previously reported evidence from a cross‐sectional analysis of the 2011 data from this cohort that noticing tobacco PoS displays more frequently when visiting shops was associated with an increased likelihood of being susceptible to smoking 11. These new prospective data demonstrate that after adjustment for the effects of other determinants of smoking behaviours, visiting shops and noticing PoS displays more frequently is associated with an increased likelihood of non‐susceptible never smokers becoming susceptible to smoking, but is not related to the likelihood of becoming an ever smoker. In addition, recognizing higher numbers of tobacco product brands was associated with an approximate twofold increase in the risk of non‐susceptible never smokers becoming susceptible to smoking or becoming an ever smoker. When we combined frequency of noticing tobacco PoS displays and number of brands recognized we found that non‐susceptible never smokers who noticed tobacco PoS displays most or every time they visited the shops and recognized six or more tobacco brands were more than three times likely to become susceptible to smoking, while these factors did not significantly influence transition to being a smoker among children who were either non‐susceptible or susceptible at baseline. We were not able to determine whether the key component of this exposure was the PoS display itself or exposure to the brands the displays contain. There was no clear explanation as to why some susceptible never smokers in 2011 became non‐susceptible in 2012. Further research with a larger sample size is necessary to investigate which factors are important to reverse smoking susceptibility.

To our knowledge, this is the first cohort study to examine changes in susceptibility to smoking among schoolchildren in relation to PoS exposure, and hence to provide insight into the probable causal direction of previously reported cross‐sectional associations between PoS exposure and smoking behaviour. As the majority of smokers take up smoking before age 18 24, and approximately 40% before age 16 25, we included children aged 11–16 to measure susceptibility to smoking, which is an important predictor of future smoking. For logistical reasons we were unable to study children aged 17 and 18. Our study population included students across a spectrum of socio‐economic disadvantage, and from rural and urban areas, so our findings are likely to be broadly representative. Although adult smoking prevalence in Nottingham is above average at 32% 26, the proportion of children in our sample who had tried smoking at least once or were current smokers in 2012 was 21.8%, which is in line with national survey data (23% in 2012) 27. However, the number of children whose susceptibility or smoking status changed during the single year of study was small, so our ability to explore differential effects of exposure in large and small retailers, and indeed the independent effects of noticing PoS displays, the frequency of visiting shops and the number of cigarette brands recognized, was limited by low study power. Therefore, to increase the power of our analyses we combined data for large and small retailers. Our findings are all based on self‐reported exposure and outcome data, and hence relatively open to error and bias; however, where possible we used measures that have previously been widely used and validated 10, 13, 27. Objective validation of exposure and outcome data was not feasible with the time and resources available.

Tobacco PoS displays are an important medium through which the tobacco industry can communicate brand imagery to children and young people 28, and also enhance the perceived popularity of tobacco products and specific brands 6. A study of adolescents' perceptions of tobacco control policies found that PoS displays were perceived to encourage smoking and cigarette purchase, and to portray smoking as attractive 29. More frequent visits to stores where tobacco products are available on PoS displays have also been shown to increase the risk of smoking uptake among adolescents 10. It is possible that the discrepancies between these and our findings arose from differences in study design (e.g. cross‐sectional versus longitudinal study design), or that the effect of PoS exposure in general is limited to increasing susceptibility, and that other factors are more important in determining progression from susceptibility to smoking experimentation.

There is a range of important factors affecting the transition from non‐susceptible to susceptible or ever smoker, such as smoking status of parents and other family members, age, subjective social status and peer smoking 14, 30, particularly among children from more deprived environments, and exposure to tobacco marketing. However, removal of PoS displays as a tobacco control policy might play an important role in reducing smoking uptake and prevalence among young people in the long term by reducing the numbers who become susceptible to smoking. Removal of PoS displays of tobacco products is widely supported by the general public 31, 32, primarily as a means to protect children from exposure to promotion of a lethal product 33. As PoS exposure also undermines the success of smoking cessation attempts 34, there is strong justification for the removal of these displays to support smokers who are trying to quit. Removal of PoS displays in Ireland led to a decrease in the proportion of adult smokers and children noticing displays, and children also thought that removal of PoS displays made it easier for children not to smoke and helped to de‐normalize smoking 32. Also, while retailers are understandably concerned that implementation of PoS display bans will reduce their income from sales of tobacco products, the effect of removal of displays on smoking prevalence, at least in the short term, is likely to be modest and have a negligible effect on sales to regular smokers 35. However, findings from our earlier work in the same cohort of children suggest that the main source of exposure to PoS displays is small shops 11, indicating that in relation to reducing uptake of smoking, ending PoS displays in small retailers is probably the more important stage of this process.

### Declaration of interests

None.

## Supporting information

Table S1 Adjusted relative risk ratios for changes in susceptibility and smoking status in relation to noticing PoS displays, frequency of visiting shops, and number of brands recognised (excluding parental, sibling and friend smoking as potential confounders).

Supporting info item
