# Supplementary material for: Exposure to point‐of‐sale displays and changes in susceptibility to smoking: findings from a cohort study of school students
Source: Addiction. 2015 Jan 20;110(4):693–702. doi: 10.1111/add.12826 (PMC4402022; doi:10.1111/add.12826)
Supplement: Supplementary file 1 — Supporting info item [file add0110-0693-sd1.docx]

Table S1: Adjusted relative risk ratios for changes in susceptibility and smoking status in relation to noticing PoS displays, frequency of visiting shops, and number of brands recognised (excluding parental, sibling and friend smoking as potential confounders)

|  | **Among non-susceptible never smokers at baseline** | | | | | | **Among susceptible never smokers at baseline** | | | | | |
| --- | --- | --- | --- | --- | --- | --- | --- | --- | --- | --- | --- | --- |
|  | **RRR of becoming susceptible^a^** | | | **RRR of becoming an ever smoker^a^** | | | **RRR of becoming non-susceptible^b^** | | | **RRR of becoming an ever smoker^b^** | | |
|  | **Estimate** | **99% CI** | **p** | **Estimate** | **99% CI** | **p** | **Estimate** | **99% CI** | **p** | **Estimate** | **99% CI** | **p** |
| **Noticing point of sale displays** | | | | | | | | | | | | |
| Sometimes or less | 1.00 |  |  | 1.00 |  |  | 1.00 |  |  | 1.00 |  |  |
| Most or every time | 1.03 | 0.96-1.11 | 0.328 | 1.8 | 0.78-4.16 | 0.069 | 2.09 | 0.85-5.11 | 0.034 | 1.3 | 0.54-3.13 | 0.434 |
| **Frequency of visiting shops** | | | | | | | | | | | | |
| Less than 2 or 3 times a week | 1.00 |  |  | 1.00 |  |  | 1.00 |  |  | 1.00 |  |  |
| At least 2 or 3 times a week | 1.39 | 1.08-1.77 | 0.001 | 1.47 | 0.57-3.76 | 0.295 | 1.19 | 0.68-2.07 | 0.430 | 1.52 | 0.95-2.46 | 0.023 |
| **Number of brands recognised** | | | | | | | | | | | | |
| None | 1.00 |  |  | 1.00 |  |  | 1.00 |  |  | 1.00 |  |  |
| 1 to 5 | 1.83 | 1.24-2.69 | <0.001 | 1.42 | 0.75-2.68 | 0.160 | 0.97 | 0.34-2.72 | 0.933 | 1.26 | 0.81-1.96 | 0.169 |
| More than 5 | 2.19 | 1.65-2.92 | <0.001 | 2.36 | 1.88-2.96 | <0.001 | 0.83 | 0.43-1.58 | 0.449 | 2.08 | 1.30-3.34 | <0.001 |
| **Combined frequency of visiting and noticing displays** | | | | | | | | | | | | |
| Visit <2/3 times per week/Notice sometimes or less | 1.00 |  |  | 1.00 |  |  | 1.00 |  |  | 1.00 |  |  |
| Visit <2/3 times per week/Notice most or every time | 2.70 | 1.34-5.45 | <0.001 | 1.88 | 0.32-11.0 | 0.356 | 1.79 | 0.48-6.68 | 0.257 | 0.84 | 0.31-2.25 | 0.652 |
| Visit >2/3 times per week/Notice sometimes or less | 2.42 | 1.07-5.46 | 0.005 | 1.33 | 0.17-10.8 | 0.722 | 0.88 | 0.11-6.91 | 0.868 | 0.93 | 0.31-2.78 | 0.855 |
| Visit >2/3 times per week/Notice most or every time | 3.22 | 1.51-6.87 | <0.001 | 2.64 | 0.49-14.2 | 0.136 | 2.03 | 0.48-8.63 | 0.206 | 1.45 | 0.53-3.97 | 0.346 |
| **Combined frequency of noticing displays and brand recognition** | | | | | | | | | | | | |
| Notice sometimes or less/0 brands | 1.00 |  |  | 1.00 |  |  | 1.00 |  |  | 1.00 |  |  |
| Notice sometimes or less /1-5 brands | 1.92 | 0.76-4.85 | 0.070 | 1.43 | 0.18-11.2 | 0.652 | 0.94 | 0.12-7.38 | 0.943 | 1.12 | 0.35-3.52 | 0.805 |
| Notice sometimes or less /6+ brands | 2.90 | 0.84-9.96 | 0.026 | 1.46 | 0.07-30.6 | 0.750 | * |  |  | 1.29 | 0.15-10.9 | 0.758 |
| Notice most or every time/0 brands | 1.80 | 0.72-4.51 | 0.101 | 1.80 | 0.47-6.94 | 0.263 | 2.89 | 0.72-11.6 | 0.049 | 1.10 | 0.35-3.46 | 0.830 |
| Notice most or every time /1-5 brands | 2.84 | 1.16-6.96 | 0.003 | 2.21 | 0.60-8.11 | 0.115 | 2.23 | 0.61-8.19 | 0.111 | 1.38 | 0.41-4.68 | 0.495 |
| Notice most or every time /6+ brands | 3.30 | 1.47-7.43 | <0.001 | 3.66 | 1.37-9.76 | 0.001 | 1.90 | 0.61-5.98 | 0.147 | 2.31 | 0.82-6.53 | 0.038 |

^a^ Adjusted for age, sex, self-perceived academic performance and rebelliousness; ^b^ Adjusted for age and sex

*Could not estimate due to small numbers
